# Real-time monitoring of a 3D blood–brain barrier model maturation and integrity with a sensorized microfluidic device†

**DOI:** 10.1039/d4lc00633j

**Published:** 2024-10-07

**Authors:** Maria Cristina Ceccarelli, Marie Celine Lefevre, Attilio Marino, Francesca Pignatelli, Katarzyna Krukiewicz, Matteo Battaglini, Gianni Ciofani

**Affiliations:** a Istituto Italiano di Tecnologia, Smart Bio-Interfaces, Viale Rinaldo Piaggio 34, 56025 Pontedera, Italy; b Scuola Superiore Sant'Anna, The BioRobotics Institute, Viale Rinaldo Piaggio 34, 56025 Pontedera, Italy; c Department of Physical Chemistry and Technology of Polymers, Silesian University of Technology, Księdza Marcina Strzody 9, 44-100 Gliwice, Poland

## Abstract

A significant challenge in the treatment of central nervous system (CNS) disorders is represented by the presence of the blood–brain barrier (BBB), a highly selective membrane that regulates molecular transport and restricts the passage of pathogens and therapeutic compounds. Traditional *in vivo* models are constrained by high costs, lengthy experimental timelines, ethical concerns, and interspecies variations. *In vitro* models, particularly microfluidic BBB-on-a-chip devices, have been developed to address these limitations. These advanced models aim to more accurately replicate human BBB conditions by incorporating human cells and physiological flow dynamics. In this framework, here we developed an innovative microfluidic system that integrates thin-film electrodes for non-invasive, real-time monitoring of BBB integrity using electrochemical impedance spectroscopy (EIS). EIS measurements showed frequency-dependent impedance changes, indicating BBB integrity and distinguishing well-formed from non-mature barriers. The data from EIS monitoring was confirmed by permeability assays performed with a fluorescence tracer. The model incorporates human endothelial cells in a vessel-like arrangement to mimic the vascular component and three-dimensional cell distribution of human astrocytes and microglia to simulate the parenchymal compartment. By modeling the BBB-on-a-chip with an equivalent circuit, a more accurate trans-endothelial electrical resistance (TEER) value was extracted. The device demonstrated successful BBB formation and maturation, confirmed through live/dead assays, immunofluorescence and permeability assays. Computational fluid dynamics (CFD) simulations confirmed that the device mimics *in vivo* shear stress conditions. Drug crossing assessment was performed with two chemotherapy drugs: doxorubicin, with a known poor BBB penetration, and temozolomide, conversely a specific drug for CNS disorders and able to cross the BBB, to validate the model predictive capability for drug crossing behavior. The proposed sensorized microfluidic device represents a significant advancement in BBB modeling, offering a versatile platform for CNS drug development, disease modeling, and personalized medicine.

## 1 Introduction

Central nervous system (CNS) disorders represent the main cause of disability and the second leading cause of death worldwide. One of the main challenges in treating CNS pathologies is that the development of innovative drugs and therapies for brain diseases is hindered by the presence of the so-called blood–brain barrier (BBB). The BBB is a continuous membrane enveloping the CNS and separating the bloodstream from the brain environment. The BBB microenvironment mainly comprises brain endothelial cells and other cellular components such as astrocytes, pericytes, microglial cells, and neurons.^1,2^ The main physiological function of the BBB is to regulate the passage of substances from the bloodstream to the CNS, such as nutrients and metabolites, while preventing the passage of potentially toxic compounds. Despite its physiological and pivotal roles, the BBB is also the main obstacle to the passage of drugs and therapeutic compounds, blocking almost 100% of large molecules and 98% of small molecules (*i.e*., below 400–500 Da in molecular weight).^3^ The ability of candidate drugs to cross the BBB and reach the brain is thus a main feature that needs to be assessed before their translation to clinical applications.

Currently, one of the most commonly used approaches involves the use of *in vivo* animal models, which has some limitations in terms of cost, time consumption, and ethical concerns.^4^ Moreover, animal models are not fully representative of the human BBB due to interspecies differences in terms of BBB conformation, and of gene and protein expression.^5^ Therefore, in recent years, several *in vitro* models of the BBB have been developed as pre-screening platforms able to provide insightful data concerning the brain-targeting efficiency of therapeutic moieties.^6,7^ Among these models, the most widely used is represented by Transwell inserts, which are porous membranes commonly exploited as a substrate for the culture of brain endothelial cells and the assessment of drug permeability.^8^ Transwell inserts are easy to set up and use; however, they lack the 3D typical organization of the cells composing the BBB, and are unable to reproduce the blood flow conditions observed in brain capillaries.^8^ Therefore, in recent years, several microfluidic devices have been developed to overcome the limitations posed by static devices.^6,7,9^ Despite the vast variety of microfluidic models currently described in the literature, there is still no definitive BBB-on-a-chip able to replicate the complexity of the *in vivo* BBB.^6,7,9^

Different configurations of BBB-on-a-chip have been proposed over the years to better mimic the physiological conditions focusing on the geometry and spatial organization of cellular components. The vertical configuration, for example, consists of two vertically aligned microfluidic channels separated by a porous membrane, the apical part of which supports the BBB. This model effectively mimics the key characteristics of the BBB by replicating cell–cell interactions and integrating flow conditions, thus surpassing the static environment of the Transwell model.^10,11^ However, this compartment arrangement does not allow for the simultaneous visualization of all involved cell types, thereby hindering comprehensive microscopic analysis. To address these limitations, planar side-by-side configurations have been proposed. This setup consists of two or more parallel horizontal microfluidic channels separated by an array of micropillars.^12,13^ Indeed, this configuration allows simultaneous imaging of cellular components while recapitulating the 3D arrangement of cells through the use of hydrogels. Despite the possibility of integrating fluidic systems, most of the models developed do not ensure direct flow contact with endothelial cells as in physiological conditions. More complex geometries such as tubular channel configuration can be achieved with self-assembly cell arrangement. They provide a more realistic model by offering, under flow conditions, a uniform shear stress distribution over the vascular compartment walls, thereby better simulating physiological conditions.^14,15^ Despite these advancements, the self-assembly of cells in the microfluidic device remains complex, especially in the vascular compartment.

Finally, the integration of a non-invasive sensorization system to monitor the BBB remains challenging in most of the configurations. Current methods aim to assess the tightness as well as maturation and integrity of biological barriers measuring the trans-endothelial electrical resistance (TEER), a physical parameter commonly exploited both *in vitro* and *in vivo*.^16^ Common approaches include the use of wire electrodes, representing an improvement over traditional chopstick electrodes.^17,18^ However, these wire electrodes can compromise sterile conditions and, above all, disrupt flow when embedded within the device. Positioning them outside the flow can prevent this issue, but increasing the distance between the BBB and the electrodes is accompanied by a reduction in measurement sensitivity.^19–21^ Thin-film electronics is a promising field, as it enables the integration of almost imperceptible electrodes close to the BBB. However, this can prove difficult depending on the manufacturing techniques used, particularly in complex geometries such as tubular channels. Finally, their design must be carefully adapted to limit their impact on microscopy imaging, as most of electrodes are not fully transparent.

In this work, we propose an innovative BBB-on-a-chip device leveraging the advantages of thin-film electronics to obtain a dynamic, physiological BBB model that can be real-time monitored optically and electrically. Non-invasive electrodes have been integrated into a planar side-by-side microfluidic circuit designed for replicating the 3D organization of cells composing the neurovascular unit (NVU), as well as for ensuring direct flow contact with endothelial cells growing in a vessel-like arrangement. After presenting the development of the sensorized microfluidic model, the BBB formed was optically and electrically characterized to confirm the system ability to accurately monitor the formation and maturation of the biological barrier. For this purpose, electrochemical impedance spectroscopy (EIS) was applied, and the results were compared with confocal imaging and permeability results, demonstrating the viability of the proposed system for distinguishing between the well-formed and poorly-formed BBB, in a quicker and non-invasive way. Moreover, EIS allowed the determination of the TEER through the use of mathematical models, in a more relevant and accurate way than standard automated equipment. Finally, a computational model was developed to set up flow conditions closely mimicking the dynamic environment of brain capillaries by estimating the wall shear stress. Based on these results, two standard chemotherapeutic drugs, doxorubicin (DOX) and temozolomide (TMZ), were tested under dynamic conditions, validating the model to predictively mimic the behaviour of the physiological BBB, thus paving the way for various applications such as the testing of new therapies for brain diseases.

## 2 Materials and methods

The microfluidic device is based on a polydimethylsiloxane (PDMS) platform placed on a coverslip glass, consisting of a perfusion channel representing the vascular domain interfacing *via* an array of trapezoidal pillars with the cerebral compartment organized into three chambers in communication through microchannels. The thin microelectrodes were patterned on the coverslip before bonding the microfluidic device with the coverslip.

### 2.1 Fabrication of the microfluidic device

The microfluidic design was drawn using AUTO-CAD software (Autodesk) and printed on Quartz Chromium photomask (Compugraphics). The fabrication process was performed through micro-replica molding (REM) by photolithography, providing a master for the implementation of soft-lithography. The master fabrication was performed on a silicon wafer of 3 inch diameter and activated *via* oxygen plasma with a plasma reactor (Colibrì, Gambetti Kenologia). A layer of 50 μm of SU8-50 photoresist (MicroChemicals, Kayaku Advance Materials) was spin-coated with a spin-coater (SPS EUROPE, Spin 150). The photoresist was soft-baked for 10 min at 65 °C and for 30 min at 95 °C, and then exposed to UV light with a MA/BA6 mask aligner (SUSS MicroTec) to transfer the design to the photoresist. The silicon wafer was post-baked for 1 min at 65 °C and at 10 min at 95 °C, developed with SU-8 Developer (MicroChemicals), and then hard-baked for 20 min at 200 °C. Once the master was fabricated, it was used to perform the replica molding with soft-lithography.

The microfluidic system was manufactured with PDMS mixing at a 10 : 1 ratio of the silicone elastomer and the crosslinking agent (SYLGARD™ 184 Elastomer Kit, DOW). The mixture was then cast on the master, degassed, and cured for 2 h at 70 °C. The PDMS replica was peeled off from the master, cut and micro-milled with a 3 mm puncher to obtain the inlets and the outlets of the channel and chambers. An additional curing was performed at 100 °C for 1 h.

### 2.2 Fabrication of the microelectrodes

The electrodes were designed using the AUTO-CAD software (Autodesk) and printed on a polyester mask (SELBA S.A.). The design was selected to incorporate six pairs of electrodes along the layer of endothelial cells, specifically two pairs for each chamber of the cerebral compartment. The sensing pads are apart 500 μm to allow placing in the middle of the array of pillars and the BBB cell units. Each sensing pad is 1 mm in length and 400 μm in width, in connection through a track of 100 μm in width with a detecting pad of 2 mm in width and 3 mm in length.

The electrode pattern was fabricated through a multi-step process involving photolithography. Initially, the clean coverslip (Epredia) underwent activation with oxygen plasma and was spin-coated with 3.5 μm negative photoresist AZ LNR-003 (MicroChemicals). The layer was soft-baked for 2 min at 120 °C, exposed to UV light with an MA/BA6 mask aligner, and post-baked for 90 s at 100 °C. Development was carried out in AZ 726 MIF Developer (MicroChemicals), followed by rinsing with deionized water. A 20 nm adhesion layer of titanium (Ti) and a 130 nm layer of platinum (Pt) were sputtered on the substrate with RF/DC magnetron sputtering (Kenosistec). The photoresist was lifted off with acetone and rinsed with isopropanol. The PDMS microfluidic device and electrode-coverslip were bonded together by oxygen plasma. The final microfluidic device was eventually baked for 20 min at 70 °C.

### 2.3 Cell cultures

Immortalized human brain microvascular endothelial cells (IM-HBMEC) provided by Innoprot were cultured under proliferation conditions using the endothelial cell medium kit (ECM, Innoprot) supplemented with 1% of heat-inactivated fetal bovine serum (FBS, Gibco), 1% of endothelial cell growth supplement (Innoprot), and 1% of penicillin/streptomycin solution (100 IU mL^−1^ of penicillin and 100 μg mL^−1^ of streptomycin, Gibco).

Human microglia HMC3 (CRL-3304, ATCC) were cultured in minimum essential medium (MEM, Gibco) supplemented with 1% of L-glutamine (100×, 200 mM, Gibco), 1% of penicillin/streptomycin solution (100 IU mL^−1^ of penicillin and 100 μg mL^−1^ of streptomycin, Gibco), and 10% of heat-inactivated FBS (Gibco).

Immortalized human astrocytes (IM-HA) provided by Innoprot were cultured with the human astrocyte growth medium kit including basal medium and growth supplement (Cell Applications, INC), supplemented with 1% of penicillin/streptomycin solution (100 IU mL^−1^ of penicillin and 100 μg mL^−1^ of streptomycin, Gibco).

Cell cultures were maintained under sterile conditions at 37 °C in a humidity-saturated atmosphere with 5% of CO_2_. The cell culture media were replaced every 48 h, and cell passaging was performed by using 0.05% trypsin–EDTA (Gibco), by using phosphate-buffered saline without Ca^2+^ and Mg^2+^ for the rinsing steps (PBS; EuroClone).

To replicate the 3D cerebral microenvironment, a co-culture of human astrocytes and human microglia was introduced into the device. Taking estimates into account, astrocytes make up about 20–40% of the brain glial cell population, while microglia make up about 10–15%: the ratio of human astrocytes to microglia was thus established at 4 : 1 to simulate the *in vivo* conditions.^22^ The cell mix was suspended in Geltrex™ LDEV-free reduced growth factor basement (Gibco), a hydrogel matrix, in a ratio of 1 : 1 in volume with a final cell density of 6 × 10^6^ cells per mL for IM-HA and 1.5 × 10^6^ cells per mL for HMC3, for a total volume of 4 μL for each parenchymal chamber. Subsequently, the co-culture suspension embedded in Geltrex™ was injected into the three parenchymal chambers. The microfluidic system was placed at 37 °C in a humidity-saturated atmosphere with 5% of CO_2_ for 10 min to enable hydrogel forming a reconstituted basement membrane. Upon the gelification, a 1 : 1 mixture of human astrocyte growth medium and MEM was supplied into the fluidic chambers through the designated inlets and outlets.

In order to mimic the vascular compartment of the BBB, IM-HBMECs were seeded in the perfusion channel at a density of 15 × 10^6^ cells per mL. The microfluidic system was flipped to induce cell adhesion on the lateral surface of the pillars and on the channel walls in proximity to the parenchymal component, thus inducing the formation of a vessel-like structure. Once the cells had firmly adhered, the perfusion channel was filled with fresh ECM medium and maintained at 37 °C in a humidity-saturated atmosphere with 5% of CO_2_. The microfluidic systems were maintained in the incubator for 5 days and the cell culture medium was changed daily.

### 2.4 Cellular assays

The cell viability in the microfluidic device was assessed using a Live/Dead cell viability assay (Thermo Fisher Scientific). After the cell culture, the cells were washed with PBS and incubated with phenol red-free medium containing 5 μg mL^−1^ of Hoechst (Invitrogen), 4 μM ethidium homodimer-1 (ThermoFisher), and 2 μM calcein-AM (Thermo Fisher Scientific) for 20 min. Following staining, the cells were washed with PBS and imaged using a confocal laser scanning microscope with a Plan Fluor 10×/0.30 objective (C2s system, Nikon).

Once the BBB model was established, the cells were fixed and stained to provide a visualization of distribution within the microfluidic system, as well as a qualitative evaluation of cell morphology and localization. The cells were fixed in a 4% paraformaldehyde solution (PFA, Sigma-Aldrich) at 4 °C for 20 min, and rinsed delicately twice with PBS. The cell membranes were permeabilized with 0.1% Triton X-100 (Sigma-Aldrich) in PBS for 20 min at room temperature and then rinsed twice with PBS. The actin filaments that compose the cytoskeleton were stained and the nuclei were stained by incubating the cells with a 10% goat serum solution in PBS containing 2.5 μg mL^−1^ of TRITC-phalloidin (Sigma-Aldrich) and 5 μg mL^−1^ of Hoechst (Invitrogen) for 1 h. After the incubation, cells were rinsed twice with PBS and then imaged with a Plan Apo Lambda 60× oil immersion objective at the confocal laser scanning microscope.

To verify the efficient maturation of the BBB, the tight junction (TJ) expression in IM-HBMECs was verified with immunofluorescence staining against zonula occludens-1 (ZO-1). The cells were fixed in a 4% PFA solution in PBS at 4 °C for 20 min, and rinsed twice with PBS. The cell membranes were permeabilized with 0.1% Triton X-100 in PBS for 20 min at room temperature, followed by a 40 min incubation in a 10% goat serum blocking solution. The cells were treated for 3 h at room temperature with primary rabbit antibody anti-ZO-1 (2.5 μg mL^−1^, Abcam) in a solution of 10% goat serum in PBS. After incubation with the primary antibody, the cells were rinsed twice with PBS and incubated for 1 h with a solution containing 10 μg mL^−1^ of secondary antibody (F(ab′)2-goat anti-Rabbit IgG (H + L) Alexa Fluor 488 conjugate, Invitrogen), 5 μg mL^−1^ of Hoechst, and 2.5 μg mL^−1^ of TRITC-phalloidin. Post incubation, the cells were carefully rinsed twice with PBS before being imaged with an S Plan Fluor ELWD 20× objective at the confocal laser scanning microscope.

### 2.5 Permeability assays

The permeability of the BBB was analyzed by assessing the diffusion of a fluorescence tracer from the vascular to the parenchymal compartment. A solution of 1 mg mL^−1^ of 70 kDa FITC-dextran (Sigma-Aldrich) in phenol red-free Dulbecco's modified Eagle medium (DMEM, Gibco) was used to conduct permeability assays. After 5 days of cell culture, the medium was aspirated from all the reservoirs. Subsequently, the dextran solution was injected into the vascular compartment, filling the perfusion channel and associated reservoirs. Simultaneously, phenol red-free DMEM cell culture medium was introduced into the remaining fluidic channels and corresponding reservoirs in the parenchymal compartment to ensure uniform hydrostatic pressures. The system was maintained at 37 °C in a humidity-saturated atmosphere with 5% of CO_2_ for 1 h. Thereafter, the medium in the parenchymal compartment was recovered and the fluorescence (*λ*_ex_ = 360 nm, *λ*_em_ = 460 nm) was measured with a Victor X3 Multilabel Plate Reader (PerkinElmer). The dextran concentration in the parenchymal compartment was calculated according to a standard calibration curve; apparent permeability coefficients were calculated using eqn (1):^23^

(1) $$P_{\text{app}}=\frac{V_{\text{rec}}\cdot \text{d}C_{\text{rec}}}{A\cdot \text{d}t\cdot C_{t=0}}$$

where *P*_app_ is the apparent permeability [cm s^−1^], *V*_rec_ is the volume recovered from the cerebral compartment [mL], d*C*_rec_ is the difference between the concentration of the dye in the cerebral compartment and in the input medium in the vascular compartment [mg mL^−1^], *A* is the surface area along which the diffusion of the dye occurs [cm^2^], d*t* is the time along which the *P*_app_ is assessed (s), and *C*_*t*=0_ is the concentration of the dye at *t* = 0 [mg mL^−1^]. The same procedure was performed for the control experiment consisting of the microfluidic device with only hydrogel matrix and cell culture medium.

### 2.6 TEER evaluation

The estimation of TEER values to assess the maturation and the integrity of the BBB was obtained by EIS using the BBB-on-chip with the integrated Pt-microelectrodes. For this purpose, TEER measurements were conducted using the EmStat4S (PalmSens), with a two-electrode configuration, between the electrode located in the vascular compartment and the corresponding one in the parenchymal compartment. Impedance spectra were recorded with PSTrace 5 software (PalmSens) using alternating current (AC) with a sinusoidal amplitude of 10 mV over a frequency range of 1 Hz to 100 kHz. EIS spectra were acquired on partial and complete BBB models to assess the contribution of each component of the system, including the electrode interface, the medium, the hydrogel, and the different cell populations, represented by electrical components such as resistors and capacitors. The considered models were: i) the chip containing just cell culture medium in the vascular compartment and hydrogel matrix in the parenchymal compartment; ii) the model i) with the addition of astrocytes and microglia in the hydrogel matrix; iii) the model ii) also including endothelial cells growing on the electrode surface. The equivalent circuits were modelled for data processing using the EIS Spectrum Analyser software to determine the adequate electrical components describing the different components and their boundary conditions. The fitting of the experimental data was performed using the Powell algorithm and the amplitude function, and was considered accurate for a deviation between experimental and calculated data lower than 2%. The TEER was extracted from the complete model considering the surface area of measurement.

### 2.7 Fluidic computational model

A computational fluid dynamics (CFD) simulation performed by exploiting COMSOL Multiphysics was conducted within the microfluidic system designed to emulate the BBB microenvironment. The simulation was performed on the volume of interest ***ℬ***, to validate the design sizes and to replicate the *in vivo* shear stress within the vessel-like endothelial cell distribution inside our device, by selecting the appropriate flow rate.

The flow was modelled as laminar, fully developed along the *y*-axis, by considering a Newtonian incompressible fluid, since the effects of stress-dependent viscosity can be neglected. The fluid was simulated as a cell culture medium at 37 °C (*ρ* = 1012 Kg m^−3^, *μ* = 0.964 mPa s),^24^ and an initial value *u*_*y*_ = 0.013 m s^−1^ on **∂*ℬ***_in_ was imposed, considering a flow rate *Q* = 40 μL min^−1^ = 6.67 × 10^−10^ m^3^ s^−1^. The steady-state condition was selected, and no-slip condition was assumed on the walls (**∂*ℬ***_walls_). The outlet condition was set *P* = 0 Pa on *ℬ*_out_, and the fluid dynamic domain was defined by a physi-controlled tetrahedral mesh.

Based on the previous considerations, the fluid dynamics is governed by the following Navier–Stokes and continuity equations (eqn (2)):^25–28^

(2) $$\left\{ \begin{array}{l} \rho (u\cdot \nabla )u=-\nabla P+\mu \nabla \cdot \left((\nabla u)+{\left(\nabla u\right)}^{T}\right)\text{ on}\;ℬ\\ \nabla \cdot (\rho u)=0\text{ on}\;ℬ\\ u=\left[0,u_{y},0\right]\;\;\text{ on}\;\partial {ℬ}_{\text{in }}\\ P=0\;\;\text{ on }\partial {ℬ}_{\text{out }}\\ u\cdot n=0\;\;\text{ on}\;\partial {ℬ}_{\text{walls }} \end{array}\right.$$

where *ρ* ∈ ℝ [kg m^−3^] is the density of the fluid, ***μ*** ∈ ℝ [Pa s] is the dynamic viscosity, ***u*** ∈ ℝ^3^ [m s^−1^] is the velocity field, *P* ∈ ℝ [Pa] is the pressure, and ***n*** ∈ ℝ^3^ is the normal vector to the surface **∂*ℬ***_walls_. Additionally, with the cell culture medium considered as a Newtonian fluid, the shear stress is proportional to the shear rate, and evaluated considering eqn (3) and (4):^27^

(3) $$\tau =\mu \left(\nabla u+{\left(\nabla u\right)}^{T}\right)$$

(4) $$\tau_{ij}=\mu {\dot{\gamma }}_{ij}$$

where ***τ*** ∈ ℝ^3×3^ [Pa] is the shear stress tensor, *τ*_*ij*_ ∈ ℝ [Pa] is the shear stress, and ${\dot{\gamma }}_{ij}\in \mathbb{R}$ is the shear rate [s^−1^].

The flow dynamics of the microfluidic system were evaluated by considering the geometry of the device, according to the computational model previously described, to replicate the *in vivo* shear stress experienced by endothelial cells.^29^ The vascular compartment was perfused using an Aladdin Syringe (AL-4000; WPI), a programmable double syringe pump with a flow rate of 40 μL min^−1^ to reproduce a shear stress of around 11–13 dyn cm^−2^, in line with physiological conditions.^30,31^

### 2.8 Drug crossing assessment

TMZ and DOX were considered as model drugs to evaluate drug permeability of the barrier. DOX was selected because of its well-known poor BBB penetration, while TMZ is able to cross the BBB and it is gold-standard for treatment of brain cancer.^32,33^ Based on the clinical pharmacokinetics, we selected a testing concentration of 10 μg mL^−1^ for both drugs.^34,35^ The microfluidic device integrating the BBB cellular components and a control without cells were compared at three different time points (15, 30, 60 min). High-performance liquid chromatography (HPLC) analysis was performed to provide quantitative results of the drugs crossing the BBB. The HPLC chromatograms were acquired on a Shimadzu Prominence HPLC equipped with a photodiode array UV-vis detector, using an Agilent TC-C18,^2^ 4.6 × 150 mm, 5 μm column.

Regarding TMZ, the mobile phase consisted of methanol (Sigma-Aldrich) and water containing 1% of HPLC-grade acetic acid (Sigma-Aldrich), for a final composition of 25% MeOH, 74% H_2_O, and 1% acetic acid. The flow rate was maintained in isometric mode at a constant flow rate of 0.5 mL min^−1^. The values of absorbance (at 328 nm) were acquired along 8 min.^36^

In the case of DOX, the mobile phase consisted of acetonitrile (Sigma-Aldrich) and water containing 1% of HPLC-grade acetic acid, for a final composition of 30% acetonitrile, 69% H_2_O, and 1% acetic acid. The flow rate was maintained in isometric mode at a constant flow rate of 0.8 mL min^−1^. The values of absorbance (at 485 nm) were acquired along 6 min.^37^

For both drugs, a sample volume of 36 μL was injected into the column, where the temperature was maintained at 25 °C to ensure a consistent separation of the compounds. The volume was extracted from the reservoirs in the parenchymal side independently from the inlets and outlet. The concentration for both drugs was estimated with respect to their calibration curves (Fig. S1†). Indeed, the linear relation was established between the peak area and drug concentration (0–10 μg mL^−1^) in phenol red-free DMEM (Gibco). Results obtained for TMZ and DOX were normalized regarding their respective controls, *i.e*., the amount of drug able to pass to the parenchymal side without the presence of the BBB.

### 2.9 Statistical analysis

All the experiments were conducted in triplicate and the resulting data analyzed using the R Software. The normality of data distribution was assessed with the Shapiro–Wilk normality test. For normally distributed data, ANOVA followed by the LSD *post hoc* test with Bonferroni correction was performed. The mean ± standard deviation error was used to represent the normally distributed data, and the statistically significant differences were reported as *p < 0.05, **p < 0.01, and ***p < 0.001.

## 3 Results

### 3.1 Sensorized microfluidic BBB model

The sensorized BBB-on-chip model developed in this work is composed of a microfluidic chip, mimicking the *in vivo* microenvironment of the BBB, directly bonded onto thin-film microelectrodes patterned on a glass substrate. The chip, made in PDMS, was manufactured through a soft-lithography process based on micro-replica molding.^38^ The intrinsic properties of the material along with the design chosen for this BBB model, the planar side-by-side configuration, enable direct microscopic cell imaging.

**Fig. 1:**
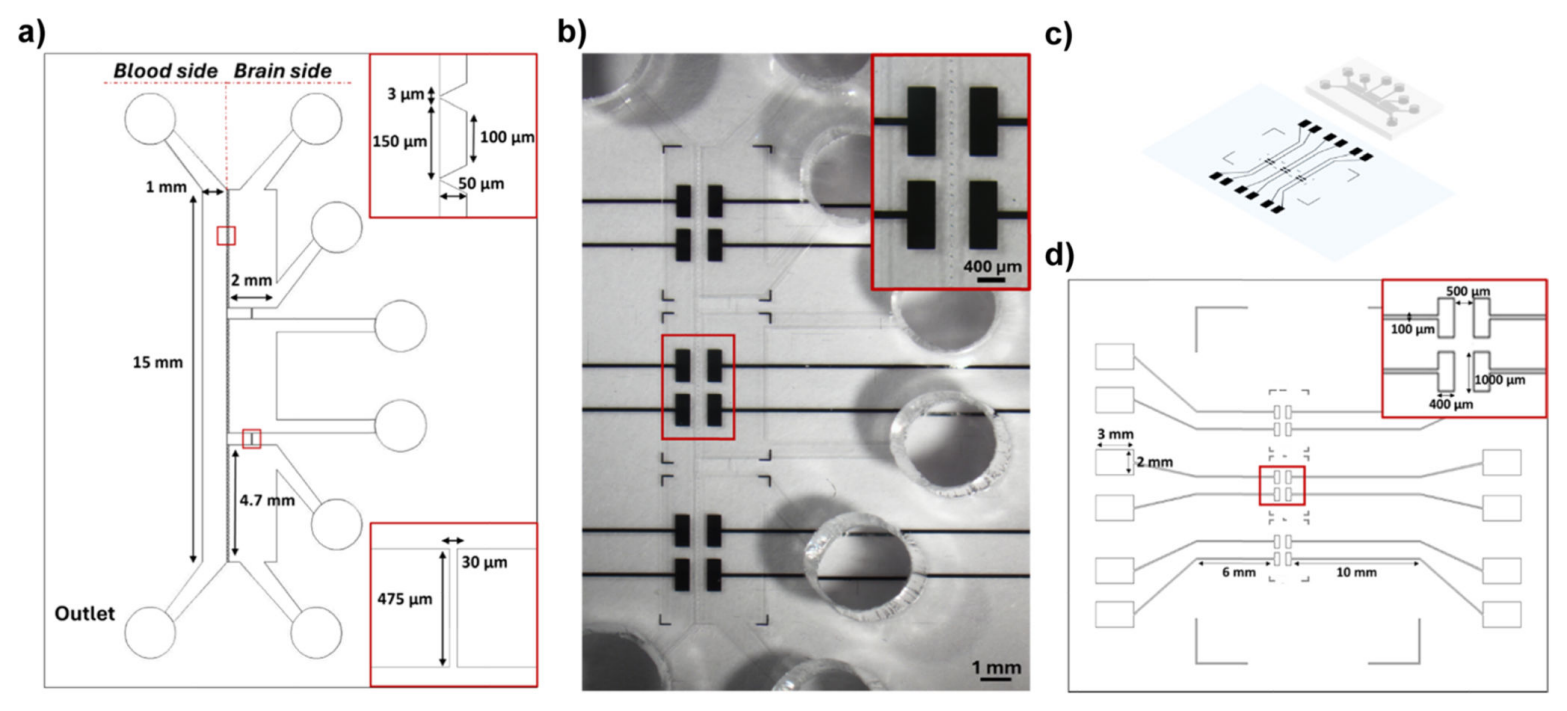
Design of the microfluidic device and of the sensorization system. (a) Schematic representation of the microfluidic device layout, detailing all the relevant sizes of the vascular compartment, represented by a perfusion channel, and of the parenchymal compartment, represented by a system of three chambers in communication *via* a microchannel; (b) optical microscopy image of the fabricated PDMS microfluidic device bonded on the glass slide with the patterned electrodes; (c) 3D rendering of the microfluidic device and the sensorization system on the glass coverslip; (d) detailed layout of the sensorization system design and sizes.

The chip consists of two microfluidic compartments: a perfusion channel representing the vascular compartment and a system of three connected chambers representing the parenchymal compartment. The geometrical features of the perfusion channel (15 mm long and 1 mm wide, for a height of 50 μm) were selected to replicate the laminar flow observed in human brain microvessels and to induce the physiological shear stress along the endothelial layer. The perfusion channel is separated from the parenchymal chambers of the same height *via* a series of trapezoidal pillars (major base of 150 μm, minor base of 100 μm), providing support for the growth of endothelial cells. The pillars are spaced 3 μm apart to confine the different cell cultures in their respective compartments during seeding, while facilitating cell–cell communication between the parenchymal and the vascular compartment once the BBB is formed. The parenchymal compartment is composed of three sub-compartments communicating through a microfluidic channel with a width of 30 μm, enabling cell migration (Fig. 1a).

In parallel, thin-film microelectrodes were patterned onto the glass coverslip using the photolithography and lift-off process. Six pairs of rectangular electrodes (400 μm wide and 1 mm long) were positioned on either side of the pillars to measure impedance across the BBB (Fig. 1b). The chosen number and sizes enable a comprehensive measurement over a broad segment at different locations of the barrier. Electrodes are made of Pt, using Ti as an adhesion layer to prevent delamination during experiments. Pt was chosen for its excellent conductivity and electrochemical properties while being inert and biocompatible.^39,40^ The final electrode thickness of 140 nm exhibited no metal delamination and did not introduce any disruptions to the flow conditions.

Finally, the PDMS chip was irreversibly bonded on the patterned microelectrodes using oxygen plasma treatment, aligning the microfluidic device with the electrodes (Fig. 1b and c). The impedance measurement can be directly performed by connecting the signal acquisition equipment to the external pads located on either side of the bonded chip, linked to the sensing area *via* conductive microtracks (Fig. 1d).

The combination of different microfabrication techniques and biocompatible materials resulted in the development of a sensorized microfluidic system mimicking the 3D microenvironment of the BBB, which can be simultaneously monitored electrically and optically over time.^38^

### 3.2 Formation of a mature BBB model

To mimic the cerebral neurovascular network, human astrocytes and microglia were embedded in a hydrogel matrix in the three parenchymal compartments of the chip, while human endothelial cells were grown in the vascular compartment along the channel walls, including the pillars.

**Fig. 2:**
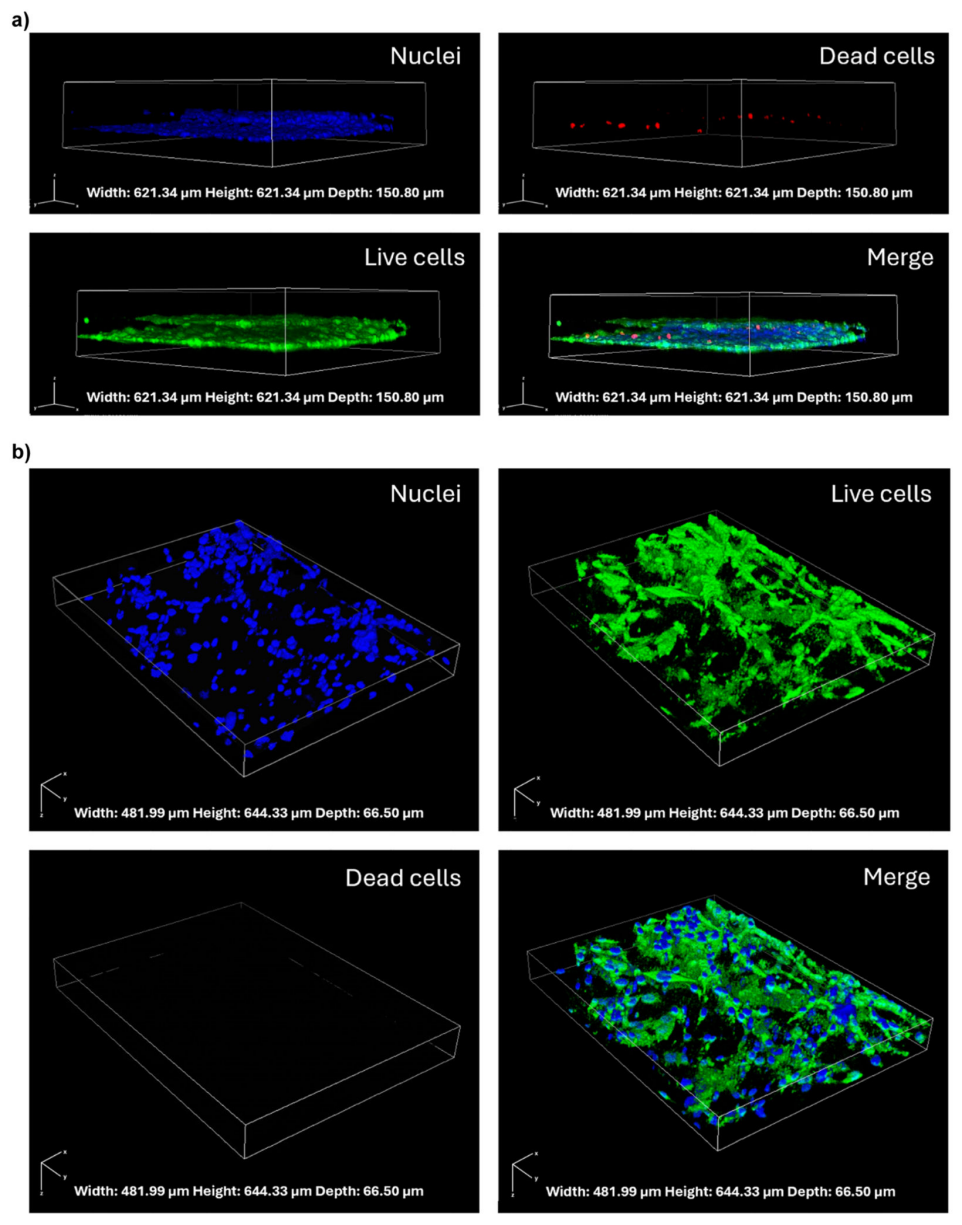
Confocal microscopy images showing live/dead cell staining and cell distribution in the microfluidic device. (a) Z-stack confocal images of the blood side of the microfluidic device showing the cell viability and vessel-like distribution of the HBMECs: nuclei (Hoechst, blue), dead cells (EthD-1, red), live cells (calcein-AM, green), and their merged view. Rendering dimension for image acquisition: width: 621.34 μm, height: 621.34 μm, depth: 150.80 μm; (b) Z-stack confocal images of the brain chambers showing a 3D arrangement of human astrocytes and microglia co-culture: nuclei (Hoechst, blue), live cells (calcein-AM, green), dead cells (EthD-1, red) and their merged view. Rendering dimension for image acquisition: width: 481.99 μm, height: 644.33 μm, depth: 66.50 μm.

After 5 days of culture, the cell arrangement and the cell viability were evaluated by the live/dead assay. On the vascular compartment, the presence of a vessel-like structure of endothelial cells was appreciated. The predominance of green fluorescence over red fluorescence indicates a high viability, with most cells maintaining their integrity, which is essential for mimicking the selective permeability of the BBB (Fig. 2a).

The parenchymal cells, a co-culture of microglia and astrocytes, exhibited a complex 3D network, closely resembling the intricate architecture of *in vivo* brain tissue, mainly located in proximity of the endothelial cell layer. The majority of the parenchymal cells were observed to be viable and healthy, indicating that the Geltrex™ matrix is capable of supporting cell survival and of promoting the three-dimensional distribution of the cells (Fig. 2b).

**Fig. 3:**
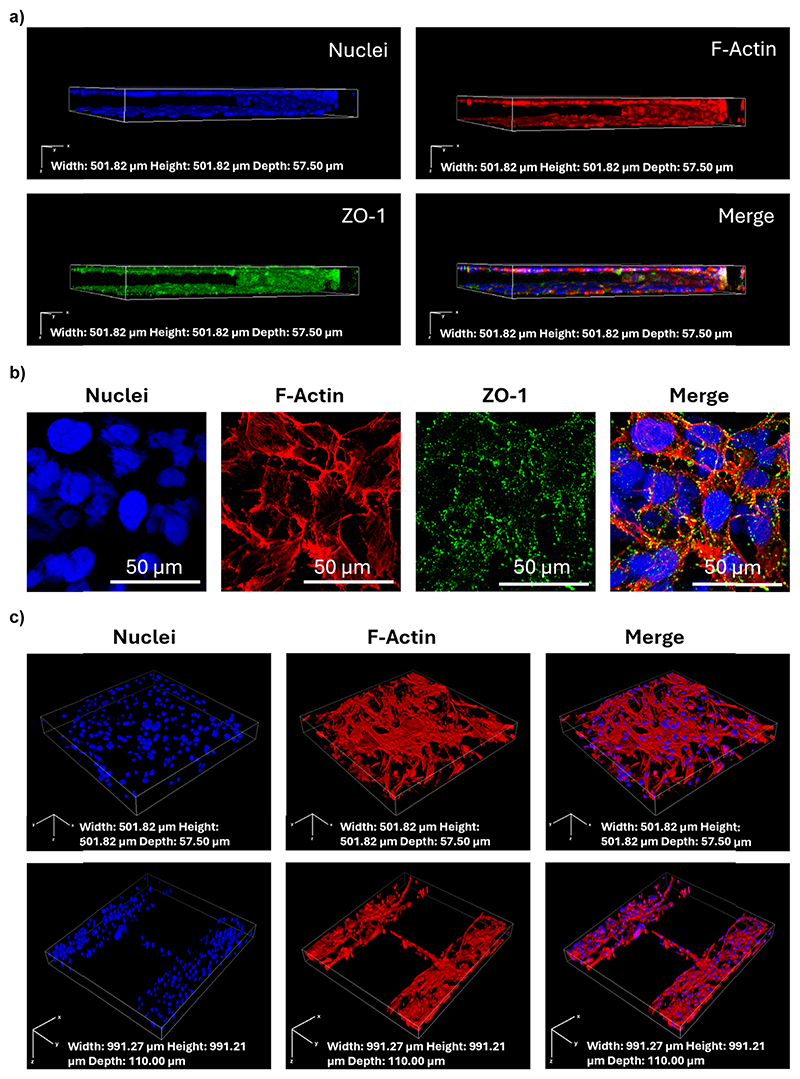
Confocal microscopy images showing immunostaining and cell distribution in the microfluidic device. (a) Z-stack confocal images of the vascular compartment of the microfluidic device showing the vessel-like arrangement of HBMECs along the perfusion channel (nuclei in blue, F-actin in red, ZO-1 in green); b) high magnification representative images of HBMECs. Rendering dimension for image acquisition: width: 501.82 μm, height: 501.82 μm, depth: 57.50 μm; (c) Z-stack confocal images of the parenchymal compartment of the device showing a 3D arrangement of the co-culture of human astrocytes and human microglia also in the microchannels connecting the three parenchymal chambers (nuclei in blue, F-actin in red). Rendering dimension for image acquisition: width: 991.27 μm, height: 991.21 μm, depth: 110.00 μm.

The maturation and integrity of the BBB model were confirmed by conducting immunofluorescence against ZO-1, a key tight junction protein expressed in the human BBB.^1^ The Z-stack confocal images show the expression and localization of ZO-1 in green, along with the cytoskeletal F-actin in red and the nuclei in blue, providing a detailed view of the cellular architecture in the vascular compartment (Fig. 3a). The continuous green staining pattern and the high magnification images (Fig. 3b) suggest that the tight junctions are well-formed and functional, a hint of a mature endothelial cell layer capable of emulating the BBB features.

The staining of the parenchymal compartment with cytoskeletal F-actin in red and nuclei in blue (Fig. 3c) showed the glial cells growing close to the endothelial cells, and a possible interaction between glial cells and endothelial cells through the 3 μm pores between pillars. The three parenchymal chambers connected *via* microchannels could support possible cell migration, as a passage of cells through the microchannels was observed (Fig. 3c).

### 3.3 *In situ* monitoring of barrier formation and maturation

The integration of thin-film electrodes enables a direct *in situ* monitoring of the BBB functionality, through EIS. This technique is based on the application of an alternating small amplitude signal (10 mV) over a wide range of frequencies (1 Hz–10 kHz) between two electrodes located on either side of the BBB model. The response signal (magnitude and phase) resulting from the perturbation of the system provides information about various electrical, electrochemical, and physical processes occurring in the system, which can be modelled with the use of an equivalent electrical circuit. In this way, it is possible to associate the changes in the charge transfer characteristics resulting from the presence of resistive and capacitive elements with the formation and maturation of the BBB.

**Fig. 4:**
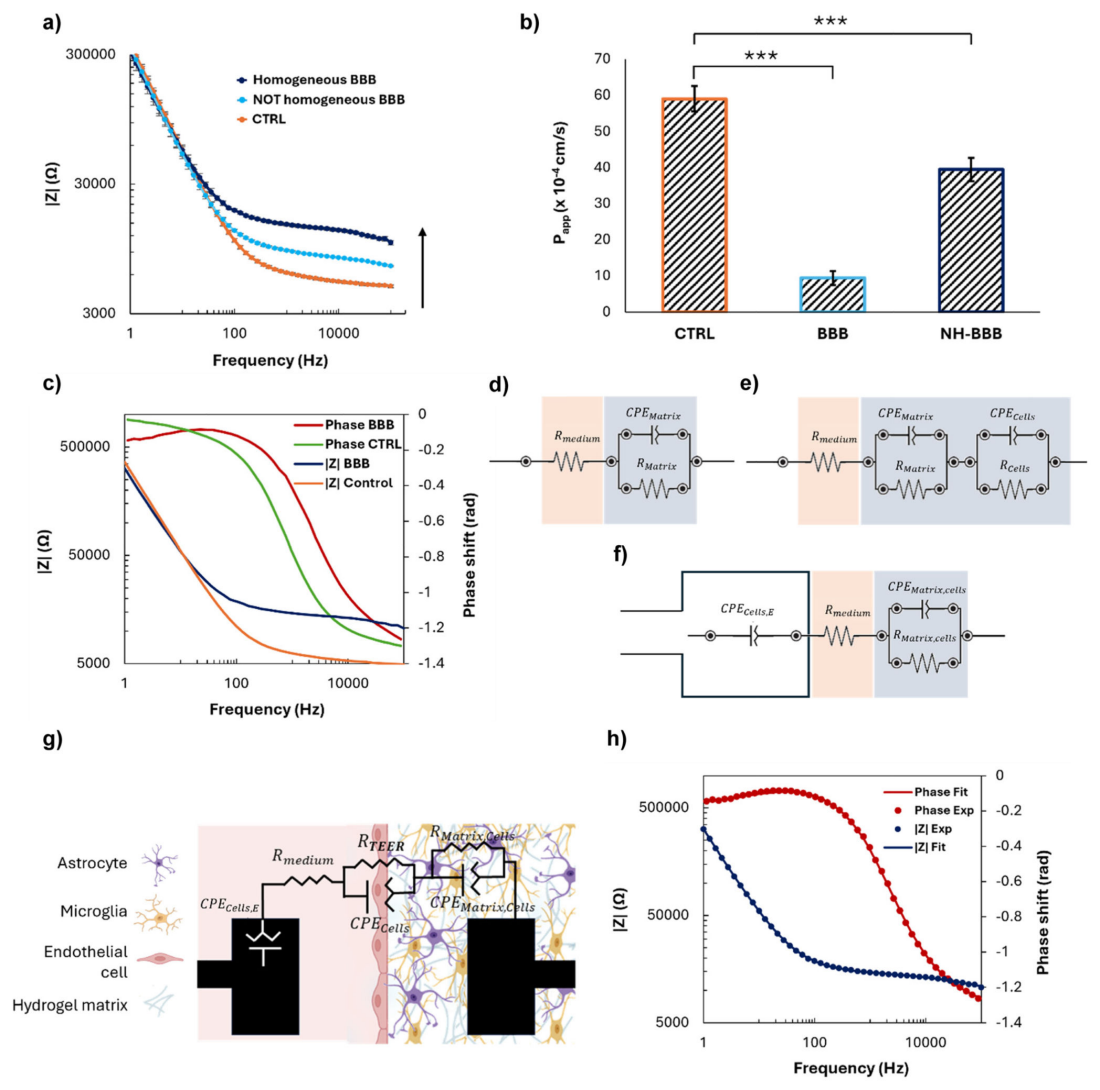
EIS analysis, permeability assessment, and TEER measurement. (a) Bode plots of impedance (|*ZI*|) *versus* frequency for the control (CTRL) and BBB models with different levels of barrier formation. The ‘Homogeneous BBB’ represents a model with a well-formed BBB and the ‘NOT Homogeneous BBB’ represents a model with an immature BBB; (b) permeability assay performed with fluorescent 70 kDa dextran (*p < 0.05, **p < 0.01, or ***p < 0.001); (c) Bode plots of phase shift and impedance (|*ZI*|) *versus* frequency for the well-formed BBB and CTRL; (d–f) equivalent circuits used to model the electrical component able to represent the BBB: modelling the hydrogel matrix component without cells (d), the hydrogel matrix component with the cells splitting the contribution of the hydrogel matrix and astrocytes and microglia (e), taking into account the cells growing on top of the electrode surface and in the hydrogel matrix (f), describing the system, where the *R*_TEER_ was extracted from (g); (h) Bode plot of phase shift and impedance (|*Z*|) *versus* frequency of the BBB model comparing the EIS spectrum obtained experimentally and the fitted one obtained from the equivalent circuit depicted on the right.

In the presence of a mature BBB, the impedance magnitude exhibited the typical frequency-dependent behavior^41^ observed across cell layers in complex environments, the amplitude of which depends on BBB maturity (Fig. 4a). At high frequencies, the signal is dominated by the resistance of the cell culture medium, while at low frequencies it is mainly associated with the capacitance arising from the accumulation of charges within the hydrogel matrix and on the cell membrane. The impedance magnitude, depicted in blue in Fig. 4a, increased for frequencies over ~50 Hz with respect to the control, in orange (*i.e*., only cell-free hydrogel and culture medium, thus representing the impedance generated by the plain device). Moreover, differences were observed in BBB models obtained under the same conditions and similar from a microscopy morphological point of view, suggesting that the biological barrier corresponding to the lowest impedance (Fig. 4a, light blue) was not fully functional and mature.

Permeability assays performed with 70 kDa FITC-dextran confirmed the results previously described, since an 80% decrease of the apparent permeability of the barrier with respect to the control was measured in the case of a mature BBB, whereas only a 30% decrease was obtained with an immature model (Fig. 4b). This confirms the ability of the sensorized device to rapidly monitor the validity of the biological model over time and in a non-invasive way, without the need for bioassays potentially altering the system integrity.

In addition to the direct *in situ* monitoring EIS provides, equivalent circuits and their corresponding mathematical models can be elaborated to analyze the behavior of the system, as well as to extract parameters such as the TEER. This approach is more relevant and precise than measuring the AC at a constant frequency using the Ohm's law, as it would neglect contributions of the capacitance of the cell layer and of the electrodes, which could lead to an overestimation of the TEER value.^42^ The phase shift observed between the control and the BBB model suggests that the system is becoming less capacitive with the addition of the cells (Fig. 4c),^42^ a consistent piece of evidence since endothelial cells are known for their resistive character. To ensure that TEER values are obtained solely from the biological barrier growing along the pillars, the equivalent electrical circuit and the corresponding boundary conditions modelling the complete BBB system were established based on the Bode spectra obtained with partial systems, and namely: i) a microfluidic platform without cells, to extract the resistance and the capacitance of the hydrogel matrix (*R*_Matrix_, CPE_Matrix_; Fig. 4d); ii) a microfluidic platform with a hydrogel matrix and brain cells, to distinguish between the resistance and capacitance of the hydrogel matrix (*R*_Matrix_, CPE_Matrix_) and brain cells (*R*_cells_, CPE_cells_; Fig. 4e); iii) a microfluidic platform with endothelial cells growing on the surface of the electrode in the vascular compartment, as well as a hydrogel and brain cells in the parenchymal compartment, with the two latter treated as a single element (*R*_Matrix,cells_, CPE_Matrix,cells_), to elucidate the effect of endothelial cells (CPE_cells,E_) on the impedance data (Fig. 4f); iv) on the complete model, where all the elements (hydrogel matrix, endothelial cells on the electrode surface, brain cells) were modelled separately to provide the final values of CPE_cells,E_, *R*_medium_, *R*_Matrix,cells_, CPE_Matrix,cells_, *R*_cells_, CPE_cells_ (Fig. 4g, Table S1†). This method allowed the contribution of the electrodes, of the hydrogel matrix, and of the different biological populations to be precisely determined, as well as setting the boundary conditions for a successful fitting.

Based on this data analysis, the final equivalent circuit was established as follows. The electrode covered with endothelial cells in the vascular part can be modelled by a so-called constant phase element (CPE), a mathematical model describing the frequency-dependence of the electrode/cell medium interface impedance, whereas a simple ohmic resistor can model the cell culture medium. Regarding the hydrogel matrix containing brain cells, and the BBB itself, they can both be modelled using a non-ideal capacitor in parallel with a resistor. In the case of the BBB, the CPE models the non-ideal capacitance of cells located at the interface between the cell culture medium and hydrogel, taking into account the possible accumulation of charges. The resistor corresponds to the TEER, relating to the resistance induced mainly by the TJs that prevent the paracellular transport of molecules and ions through the BBB. The accuracy of this equivalent circuit was confirmed by fitting the experimental data (Fig. 4h) with the corresponding mathematical model, which exhibited a deviation lower than 2%. Finally, the *R*_TEER_ was normalized for the surface area of measurement (2.35 × 10^−3^ cm^2^) and the TEER value was extracted, obtaining 17.83 ± 1.61 Ω cm^2^, a value in the range of similar BBB-on-chip models.^43^

### 3.4 Fluidic modeling

The CFD simulation was conducted with the aim of assessing the fluid behavior within the microfluidic system, with a particular focus on the distribution of velocity and wall shear stress within the perfusion channel, representing the vascular component. The flow rate, 40 μL min^−1^, was selected based on the geometry of the microfluidic system considering the simplified flow-dependent shear stress equation for a rectangular channel, to simulate the *in vivo* shear stress confirmed by the CFD results.^44^

**Fig. 5:**
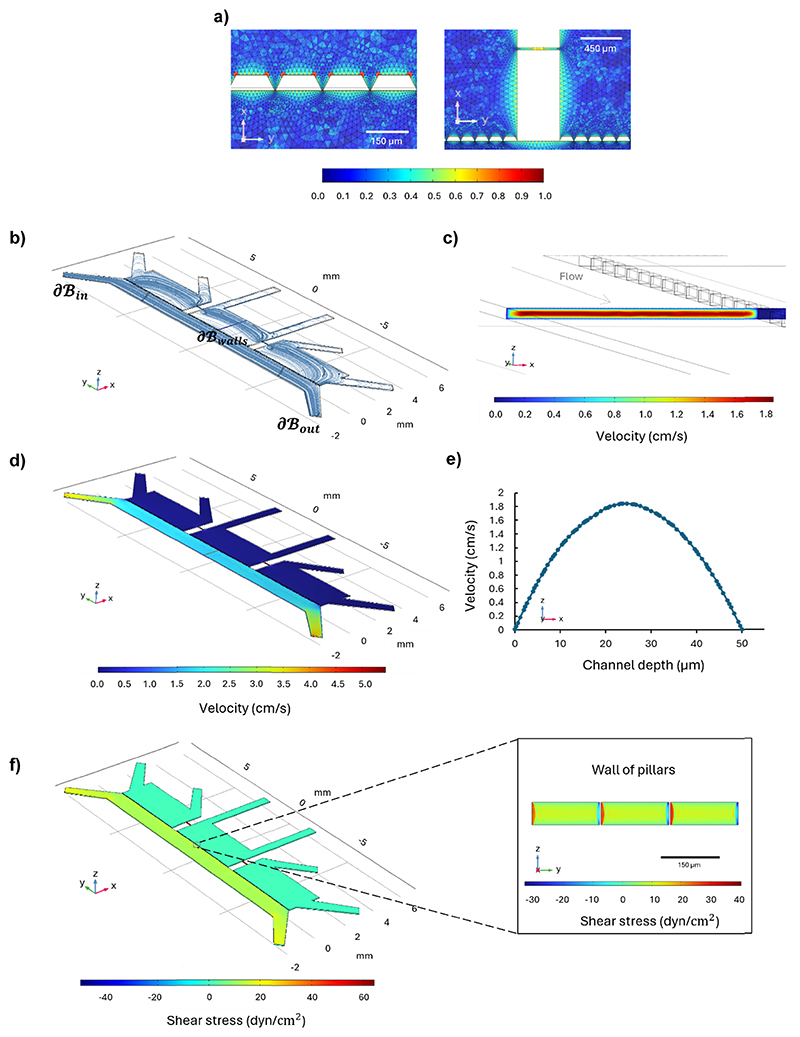
Computational fluid dynamics (CFD) simulations illustrating fluid behavior within the microfluidic system at a flow rate of 40 μL min^−1^. (a) Close-up views of the mesh around the pillars and in the microchannels between the parenchymal chambers; (b) streamline plot demonstrating parallel fluid movement along the perfusion channel in the *xy*-plane at a height of (25 μm), equal to half the total height of the channel (50 μm); (c) velocity magnitude distribution, showing a maximum velocity of 1.84 cm s^−1^ at the center and a minimum of 0.00792 cm s^−1^ at the walls in the *xz*-plane; (d) velocity magnitude distribution throughout the microfluidic device in the *xz*-plane at 25 μm; (e) parabolic velocity profile across the channel width, indicating laminar flow in accordance with a Reynolds number above 0.07; (f) shear stress distribution on the channel walls in the *xz*-plane and along the pillar walls in the *yz*-plane where endothelial cells grow, revealing a wall shear stress values of approximately 11 dyn cm^−2^ at the channel center and an average of 13 dyn cm^−2^ at the pillar walls.

On the volume of interest ***ℬ***, a mesh controlled by physics with 1 786 280 elements ensured grid-independent results, as reported in the color mesh map detailing the mesh around the pillars and in the microchannels between the brain chambers (Fig. 5a). The fluid distribution represented by the streamline plot (Fig. 5b) showed that the fluid moves parallel to the perfusion channel; Fig. 5c shows the highest velocity magnitude of 1.84 cm s^−1^ in the center of the channel, and a minimum velocity of 0.00792 cm s^−1^ at the walls due to the no-slip condition, while Fig. 5d shows the velocity distribution throughout the microfluidic device in the *xz*-plane at *z* = 25 μm. The velocity profile across the channel width is parabolic, as expected for a laminar flow and confirmed by the Reynolds number below 0.07 (Fig. 5e). Wall shear stress was evaluated on the walls of the perfusion channel and along the wall of the pillars, where the endothelial cells grow, as reported in Fig. 5f. The wall shear stress on the upper and bottom walls of the perfusion channels is around 11 dyn cm^−2^ in the center, while at the center of the wall of pillars it is around 13 dyn cm^−2^, values in agreement with the shear stress sustained by the endothelial cells under *in vivo* conditions (Fig. 5f).^30,31^

### 3.5 Drug crossing tests

The microfluidic device was further validated under dynamic conditions by evaluating the permeability of the barrier to two chemotherapy drugs. The evaluated drugs were TMZ and DOX; TMZ is a chemotherapy drug known for its ability to cross the BBB,^45^ while DOX has limited BBB permeability.^46,47^

**Fig. 6:**
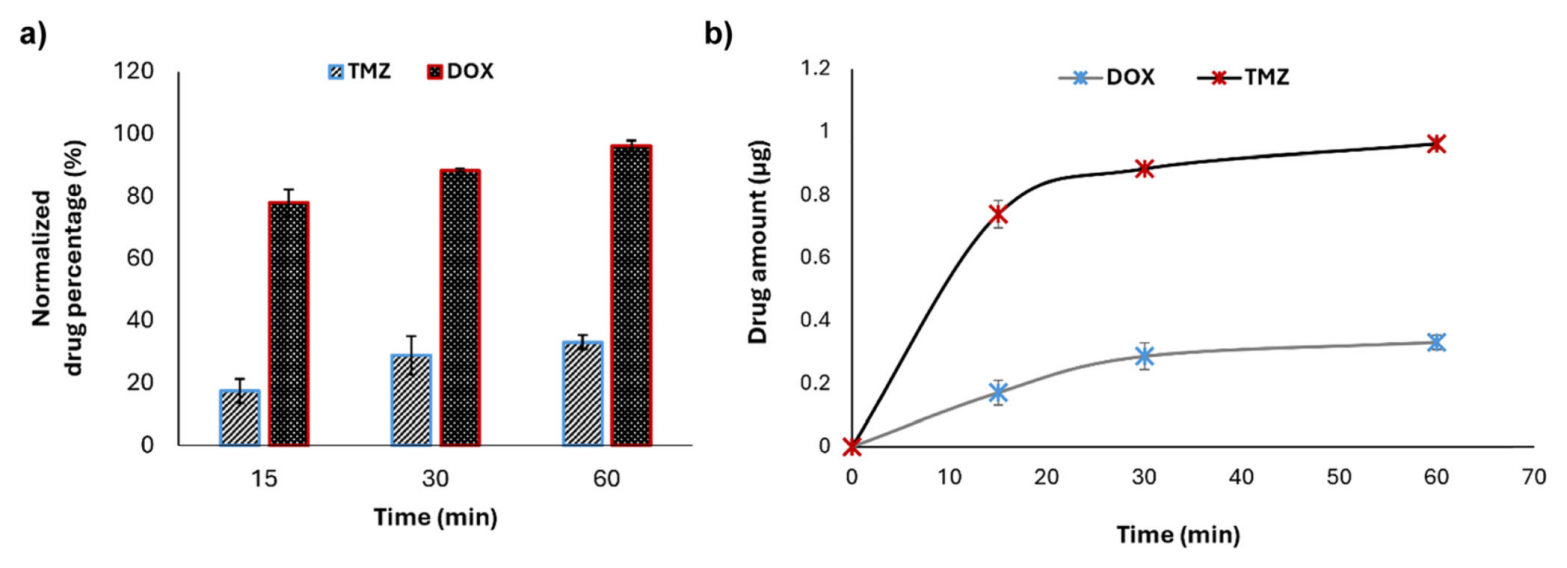
Evaluation of drug crossing through the BBB-on-chip. (a) Permeability assessment for TMZ (temozolomide) and DOX (doxorubicin) at a concentration of 10 μg mL^−1^ under continuous flow; (b) amounts of drugs crossing the BBB over time.

Cellular media containing TMZ and DOX at a concentration of 10 μg mL^−1^ were continuously infused into the microfluidic chip to simulate the actual shear stress occurring in capillaries *in vivo*. The results indicated that TMZ exhibits a significantly higher permeability across the BBB with respect to DOX, thereby confirming the model capability to distinguish between drugs with different BBB permeability profiles. In particular, TMZ showed a normalized drug permeability of about 78% at 15 min, 88% at 30 min, and 96% after 60 min in flow, while, at the same time points, DOX showed a permeability of about 17%, 28%, and 33%, respectively (Fig. 6a). The passage of drugs, although at different extents, is time-dependent and a plateau is reached after 60 min (Fig. 6b). At the end of the experiment, the impedance was measured by EIS to evaluate TEER alteration, and no change was highlighted (before the flow: 17.83 ± 1.61 Ω cm^2^; after the flow: 18.74 ± 0.32 Ω cm^2^), confirming the integrity of the BBB and suggesting the suitability of the device to predict drug crossing behavior consistently with their actual pharmacokinetics (Fig. S2†).

## 4 Discussion

This study presents a novel sensorized microfluidic device that enables real-time monitoring of BBB maturation and integrity, addressing critical challenges in CNS drug discovery. By incorporating a 3D cell arrangement and a flow directly in contact with the endothelial layer in a vessel-like configuration, our device effectively mimics the *in vivo* BBB microenvironment, providing a more physiologically relevant model compared to traditional Transwell inserts and other static *in vitro* models. The device fabrication process involves a microfluidic chip made from a biocompatible and transparent polymer (PDMS) directly bonded onto thin-film microelectrodes patterned on a glass substrate. This design choice facilitates detailed observation and better replication of the BBB complex cellular interactions.

A key innovation of the proposed device is the integration of thin-film electrodes for *in situ* measurement of TEER using EIS. This approach enables non-invasive, real-time monitoring of BBB integrity, offering a significant advantage over traditional methods that rely on endpoint measurements. The observed increase in TEER over time confirms the successful formation and maturation of the BBB within the device, demonstrating its potential for dynamic studies of BBB functionality. The TEER values obtained from our device were found to be consistent with those previously reported in analogous BBB-on-a-chip systems (19–37 Ω cm^2^),^43,48^ thereby validating the efficacy of the approach. The discrepancy between the TEER values obtained with the described *in situ* sensorization system (~19 Ω cm^2^) and some of those obtained with other *in vitro* models (50–4000 Ω cm^2^)^21,49,50^ can be attributed to several factors such as the device configuration, the type of cell, and the porosity and coating of the membrane and electrodes exploited for measurement, among others. In particular, the TEER values extracted in a similar model that integrates Ag/AgCl electrode wires in a sandwich configuration with a porous membrane were higher (150 Ω cm^2^), which could be attributable to the nature of the electrodes and to their positioning. Indeed, they are inserted at the end of the inlet and outlet of the channels, and the presence of the fibronectin-coated polycarbonate membrane can increase the resistance.^10^

Thin microelectrodes were introduced in a planar configuration by Rissanen *et al*.^51^ for monitoring capillary endothelial cell culture and then recently by Palma-Florez *et al*. for TEER measurement.^52^ The TEER value extracted by EIS with gold electrodes approached 4000 Ω cm^2^ but was integrated into different cell lines, and considered different electrical components to model the equivalent circuit.^52^ In addition, the TEER discrepancies compared to Transwell systems can be primarily attributable to the methodology employed for the detection. TEER values measured with chopstick electrodes such as the epithelial voltohmmeter (EVOM) or its upgraded versions the REMS AutoSample or Millicell-ERS system tend to be higher than those obtained using EIS due to several factors. Chopstick electrodes can produce non-uniform current density across the cellular monolayer; moreover, the TEER is measured at just one frequency (usually 12.5 kHz), which can lead to an overestimation of the values.^49,53^ Conversely, EIS offers a more precise estimation by separating different impedance contributions using a frequency sweep, leading to lower but more accurate TEER values. The BBB model proposed in this work was thus accurately characterized by analyzing the impedance spectrum, distinguishing the various cell populations and the hydrogel matrix, and removing the capacitive contribution of cells growing on the top of the electrode surface. This ensured that the TEER was mainly related to the endothelial layer growing along the pillars, thus providing a more nuanced understanding of BBB maturation and integrity with respect to traditional single-frequency TEER measurements.^42^

The controlled flow conditions, mimicking *in vivo* shear stress, support an accurate assessment of drug permeability, crucial for evaluating the potential of therapeutic agents to cross the BBB. Testing standard chemotherapeutic drugs, DOX and TMZ, under dynamic conditions demonstrated that the model predictively mimics the behavior of such compounds *in vivo*, paving the way for future applications in CNS drug development.

The CFD simulation assessed fluid behavior within the microfluidic system, focusing on the distribution of velocity and wall shear stress within the perfusion channel. The results showed a parabolic velocity profile and wall shear stress values consistent with *in vivo* conditions, confirming that the device accurately simulates the physiological shear stress experienced by endothelial cells. This aspect is crucial for ensuring the relevance of drug permeability studies.

The sensorized model presented in this work is highly versatile, and can be adapted to various applications. Regarding pharmacological studies, alternative polymers might be considered as studies demonstrated potential absorption of drugs onto PDMS.^54^ To overcome this limitation, different coatings can be performed on PDMS to reduce the absorption of small molecules.^55^ Poly(methyl methacrylate) (PMMA) and polycarbonate (PC) present moreover a compelling alternative due to their lower drug absorption rates compared to PDMS, with PMMA retaining excellent optical clarity while PC is slightly lower than PDMS and PMMA.^54,56^ However, both PMMA and PC have a hydrophobic surface that necessitates surface treatments to enhance cell adhesion, as well as fabrication and bonding processes, which are more tricky. Regarding the sensorization system, one might consider the incorporation of transparent electrodes such as graphene electrodes to further enable real-time monitoring capabilities while maintaining optical clarity for microscopy imaging for each point of the BBB.^57^ Moreover, cellular arrangements in the chip can be made more complex by involving organoids or spheroids to replicate the 3D cell organization in the brain, and provide a more comprehensive model of the neurovascular unit. Personalized medicine studies can be facilitated through the integration of patient-derived cells into the model, allowing for the study of individual patient responses to therapeutic agents.^58^ Additionally, the versatile design enables its utilization for cancer-on-chip models as well, by inserting different cell lines into the brain compartment such as cancer cells and immune cells to evaluate their interaction by the communication microchannel allowing cell migration.

## 5 Conclusion

The proposed sensorized microfluidic device represents a significant advancement in BBB modeling, offering real-time, non-invasive monitoring of BBB integrity and permeability in physiologically and pathologically relevant environments. This platform holds great promise for drug screening, disease modeling, and personalized medicine, providing a valuable tool for advancing our understanding of the BBB and developing new therapies for CNS disorders.

## Data Availability

The data that support the findings of this study are openly available in Zenodo at https://doi.org/10.5281/zenodo.12804228.^59^
