# Supplementary material for: Real-time monitoring of a 3D blood–brain barrier model maturation and integrity with a sensorized microfluidic device
Source: Lab Chip. 2024 Oct 7;24(22):5085–100. doi: 10.1039/d4lc00633j (PMC11482549; doi:10.1039/d4lc00633j)
Supplement: Supplementary file 1 [file LC-024-D4LC00633J-s001.pdf]

Supplementary Information (SI) for Lab on a Chip.
This journal is © The Royal Society of Chemistry 2024

SUPPORTING INFORMATION

Real-time monitoring of a 3D blood-brain barrier model maturation and integrity with a sensorized microfluidic device

Maria Cristina Ceccarelli[1,2], Marie Celine Lefevre[1], Attilio Marino[1], Francesca Pignatelli[1], Katarzyna Krukiewicz[3], Matteo Battaglini[1]; Gianni Ciofani[1*]*

[1]Istituto Italiano di Tecnologia, Smart Bio-Interfaces, Viale Rinaldo Piaggio 34, 56025 Pontedera, Italy

[2]Scuola Superiore Sant'Anna, The BioRobotics Institute, Viale Rinaldo Piaggio 34, 56025 Pontedera, Italy

[3]Silesian University of Technology, Department of Physical Chemistry and Technology of Polymers, Księdza Marcina Strzody 9, 44-100 Gliwice, Poland

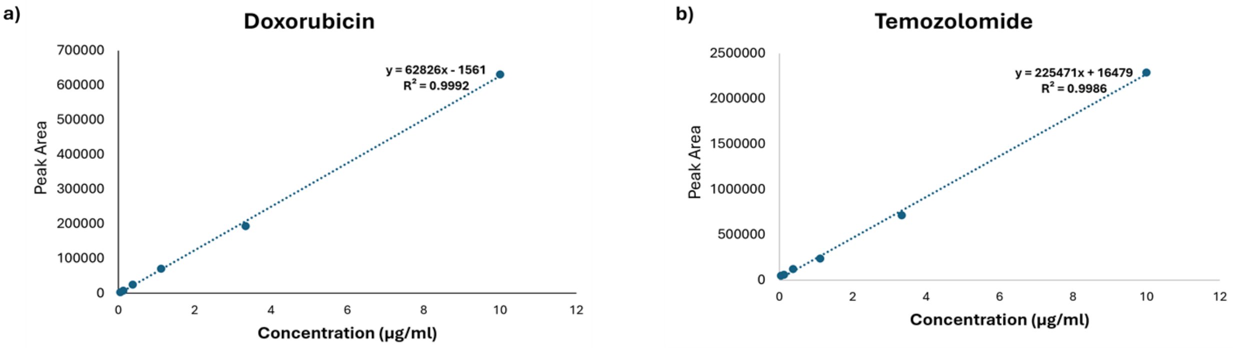

Figure S1. *HPLC calibration curves for doxorubicin (a) and temozolomide (b).*

Parameters	Values	Units
R_{TEER}	7588.13 ± 688.54	Ω
R_{medium}	7035.90 ± 943	Ω
$R_{matrix,cells}$	3.10 ± 5.00 10^{10}	Ω
$P_{cells,E}$	8.90 ± 0.61 10^{-7}	-
$n_{cells,E}$	0.78 ± 0.01	-
$P_{matrix,cells}$	3.30 ± 1.85 10^{-6}	-
$n_{matrix,cells}$	0.94 ± 0.05	-
P_{cells}	1.80 ± 0.43 10^{-7}	-
n_{cells}	0.502 ± 0.004	-

Table S1. *Parameters extracted by fitting the EIS spectrum with the equivalent circuit considering the model, taking into account endothelial cells in the vascular component and the co-culture of astrocytes and microglia in the hydrogel matrix in the parenchymal compartment.*

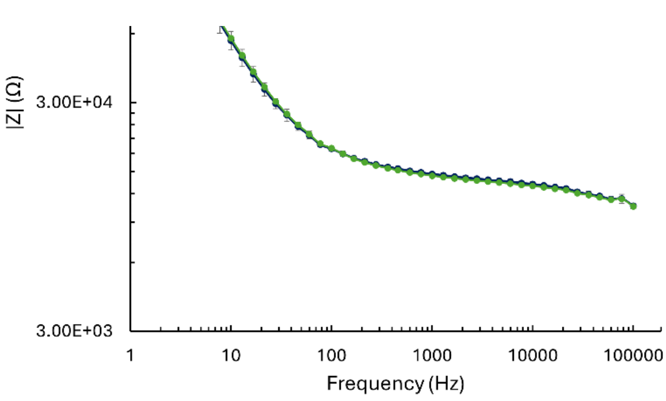

Figure S2. *Bode plots of impedance (|ZI|) versus frequency for the control BBB model before and after the drug crossing experiment.*
